# A multicenter randomized trials to compare the bioequivalence and safety of a generic doxorubicin hydrochloride liposome injection with Caelyx ^®^ in advanced breast cancer

**DOI:** 10.3389/fonc.2022.1070001

**Published:** 2022-12-20

**Authors:** Yinjuan Li, Lu Qi, Yu Wang, Yan Li, Chunpu Lei, Yingjuan Zhang, Xiaoqiang Cheng, Ju Liu, HaiHong Bai, Xia Zhao, Shuzhen Lv, Bingjun Xiong, Juan Liu, Yehui Shi, Huan Zhou, Hongtao Li, Lihong Liu, Hongchuan Jiang, Weiwei Ouyang, Xiaowen Li, Yanping Li, Xinghe Wang

**Affiliations:** ^1^ Department of Phase I Clinical Trial Center, Beijing Shijitan Hospital, Capital Medical University, Beijing, China; ^2^ Department of Breast Surgery, Beijing Shijitan Hospital, Capital Medical University, Beijing, China; ^3^ Department of Phase I Clinical Trial, Tianjin Medical University Cancer Institute and Hospital, Tianjin, China; ^4^ National Institute of Drug Clinical Trial, The First Affiliated Hospital of Bengbu Medical College, Bengbu, China; ^5^ Oncology Department, The First Affiliated Hospital of Bengbu Medical College, Bengbu, Anhui, China; ^6^ Pharmacy Department, Beijing Chaoyang Hospital, Capital Medical University, Beijing, China; ^7^ Department of Breast Surgery, Beijing Chaoyang Hospital, Capital Medical University, Beijing, China; ^8^ Oncology Department, The Affiliated Cancer Hospital of Guizhou Medical University, Guiyang, China; ^9^ Clinical Medical Center General Manager Shanghai Fudan-Zhangjiang Bio-Pharmaceutical Co., Ltd., Shanghai, China

**Keywords:** bioequivalence, pegylated liposomal doxorubicin (PLD), pharmacokinetics, breast cancer, free doxorubicin, encapsulated doxorubicin

## Abstract

**Purpose:**

To compare the pharmacokinetic (PK) bioequivalence (BE) and safety of a generic pegylated liposomal doxorubicin (PLD) formulation with the reference product Caelyx^®^.

**Methods:**

A multicenter, single-dose, open-label, randomized, two-way crossover study was conducted in patients with breast cancer. For each period, the patients were administered with the test or the reference PLD intravenously at a dose of 50 mg/m^2^. C_max_, AUC_0−t_ and AUC_0−∞_ for free, and encapsulated doxorubicin (doxorubicin) and partial AUC (AUC_0−48h_, AUC_48h−t_) for encapsulated doxorubicin were evaluated in 17 blood samples taken predose, and increasing time intervals over the following 14 days in each period. A washout period of 28-35 days was observed before crossing over.

**Results:**

48 patients were enrolled and randomised, of which 44 were included and analysed in bioequivalence set (BES). The 90% confidence intervals (CIs) of the geometric mean ratio (GMR) of C_max_, AUC_0−t_ and AUC_0−∞_ for free doxorubicin and encapsulated doxorubicin all fall within the bioequivalent range of 80% to 125%. The 90% CIs of GMR of partial AUC (AUC_0−48h_, AUC48_h−t_) for encapsulated doxorubicin also fall within the bioequivalent range. 48 patients were all included in the safety set (SS). The incidence of treatment-emergent adverse events (TEAEs) related to T and R was 95.8% (46/48) and 97.8% (45/46) respectively. The highest incidence of TEAEs was various laboratory abnormalities. 2 patients withdrew due to T-drug-related AEs. Only one patient experienced serious adverse events and no death occurred in this study. There were no significant differences between the safety profiles of the generic formulation and Caelyx^®^.

**Conclusions:**

Bioequivalence between the test and the reference products was established for free and encapsulated doxorubicin.

**Clinical trial registration:**

http://www.chinadrugtrials.org.cn, identifier [CTR20210375].

## Introduction

Breast cancer is the most common malignancy in women, about 2.26 million new cases occur worldwide each year, accounting for 24.5% of all new cancers in women (1, 2). According to the American Cancer Society 2021, breast cancer is the leading cause of cancer death among women aged 20-59 (3).

Doxorubicin (doxorubicin) hydrochloride (HCl) is a cytotoxic medicine that belongs to the group ‘anthracyclines’. It interferes with the DNA in cells, preventing them from making more copies of DNA and making proteins (4, 5). It has been written into NCCN breast cancer guidelines and other anti-tumor treatment guidelines. The clinical application of conventional anthracyclines will be accompanied by adverse reactions such as cardio-toxicity, bone marrow suppression and hair loss. The most toxic reaction is the cardio-toxicity, which is the dose limiting toxicity of anthracyclines, causing progressive irreversible congestive heart failure. This failure is dose-dependent, when the dose exceeds 450–550 mg/m^2^ and there is no effective prevention or treatment (6, 7). Moreover, the apparent volume of distribution (V_d_) is quite large, ranging from 20-30 L/kg, suggesting an extensive tissue uptake (8). These shortcomings limit the clinical application of anthracyclines.

Pegylated liposomal doxorubicin (PLD) is doxorubicin HCl encapsulated in liposomes with surface-bound methoxypolyethylene glycol (MPEG). This process is known as pegylation and protects liposomes from detection by the mononuclear phagocyte system (MPS), which increases blood circulation time (7, 9). In addition, the average particle size of liposomal doxorubicin is 90nm (called nano drug). Due to the irregular expansion of micro-vessels in tumor tissue, the vascular endothelial cells are loose and discontinuous, and the gap may up to 100nm-2μm, it is easy for liposomal drugs to penetrate into tumor tissue from local blood vessels, and the lack of functional lymphatic drainage in tumor tissue resulting in passive enrichment and accumulation of liposomal drugs in tumor tissue, which is called enhanced permeability and retention (EPR) effect (4, 7, 9, 10). The drug concentration in tumor tissue is high, and the drug distribution in other healthy tissues and organs is reduced, so as to improve the anti-tumor efficacy and reduce adverse reactions. Studies showed that the concentration of PLD in tumor tissue was 20-60 times compared to healthy tissues, and the half-life can be as long as 73.9h, while the half-life of doxorubicin was less than 10min (11, 12). Through passive targeting, PLD can minimize the cardio-toxicity of traditional doxorubicin on the basis of ensuring clinical efficacy.

PLD injection was first approved in US in1995 as Doxil^®^, followed by marketing authorization in Europe in 1996 under the Caelyx^®^ brand name. It is indicated for the treatment of metastatic breast cancer in patients at risk of heart problems, Kaposi’s sarcoma in patients with AIDS, recurrent ovarian cancer and multiple myeloma (6, 13).

With growing needs for affordable and effective anticancer treatments, the development of generics is becoming increasingly important to facilitate patient access to and affordability of vital medications. However, unlike most cases of small molecule generic products that enter the market soon after the originator product’s patents and exclusivities expire, in spite of the long-marketing history of Doxil^®^, there are few approved generic drugs available (14), which boils down to technical hurdle for the formulation of the liposomal preparation and the bioanalysis for preclinical and clinic studies (10, 14, 15). The formulation process of a liposome preparation consists of multiple physical-chemical steps including hydration, sizing, and removal of free drugs (15). Even minor changes in the formulation or manufacturing process may alter their distribution characteristics *in vivo* performance. Even if liposomal preparations have the same physical and chemical properties, they may show different efficacy and toxicity characteristics. Therefore, these preparations are more difficult to copy than small-molecule medications.

Shanghai Fudan-Zhangjiang Bio-Pharmaceutical Co., Ltd., China, has developed a PLD injection (Trade name: libaoduo, specification: 20 mg/10 ml) as a generic version of Caelyx^®^. The objective of this study was to assess the PK bioequivalence between the PLD injection manufactured by Shanghai Fudan-Zhangjiang Bio-Pharmaceutical Co., Ltd. and the reference product (Caelyx^®^) and compare the safety of the two products in patients with advanced breast cancer after a single dose administration (50 mg/m^2^ dose).

## Materials and methods

### Study design

This multicenter, open-label, two-treatment, two-period, two-sequence, single-dose, two-way crossover, bioequivalence study was conducted at 5 centers across China between March 2021 and December 2021. The study protocol was approved by the institutional ethics committee of Beijing Shijitan Hospital affiliated to Capital Medical University. The study was conducted in accordance with the Declaration of Helsinki and the requirements of local regulations in China and ICH-GCP guidelines. This trial has been registered in the Drug Trial Registration and Information Publication Platform (chinaDrugtrials.org.cn) as CTR20210375.

### Patient population

Patients aged 18 to 75 years old were eligible to participate if they had a histological or cytological diagnosis of breast cancer and expected to benefit from doxorubicin liposome mono-therapy. Eligibility criteria included the following: life expectancy of at least 3 months; Eastern Cooperative Oncology Group (ECOG) performance status (PS) ≤2; adequate bone marrow function [absolute neutrophil count (ANC) ≥1,500/mm^3^, hemoglobin ≥9 g/dl, and platelet count ≥80,000/mm^3^]; adequate renal function (serum creatinine ≤1.5× Institutional upper limit of normal (ULN)); adequate coagulation function [prothrombin time (PT), activated partial thromboplastin time (APTT) ≤1.5×ULN]; adequate hepatic function [aspartate aminotransferase (AST), alanine aminotransferase (ALT) level ≤2.5×ULN, and total bilirubin level ≤ULN]; and no active central nervous system (CNS) metastases, and normal left ventricular ejection fraction (LVEF ≥ 50%) by echocardiogram. Patients may not have had chemotherapy or radiotherapy within 28 days of starting the protocol treatment. Patients with prior doxorubicin exposure that would result in a total lifetime exposure of 550mg/m^2^ or more after four periods of treatment were excluded. Patients must have provided signed and dated informed consent forms.

### Study products and administration

**Figure 1:**
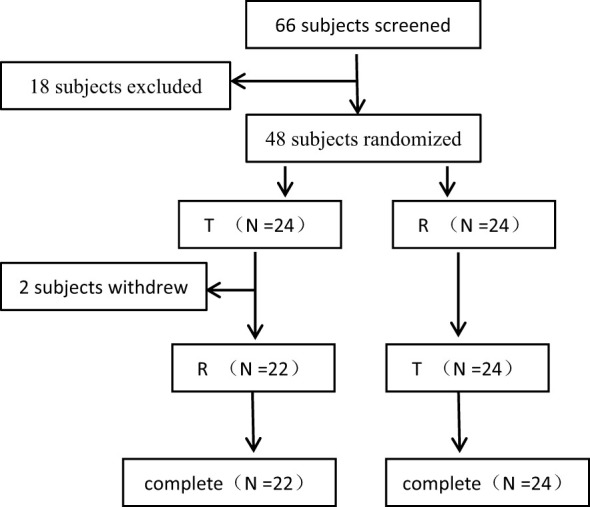
The Flowcharts of the Subject Distribution.

The reference (R) formulation was Caelyx^®^ (specification: 20 mg/10 ml, Janssen-Cilag International NV, Belgium), and the test (T) formulation was PLD injection developed by Shanghai Fudan-Zhangjiang Bio-Pharmaceutical Co., Ltd., China (Trade name: libaoduo, specification: 20 mg/10 ml). Both the T and R formulation were administered by intravenous infusion at a single dose of 50 mg/m^2^ once every 4 weeks according to the randomization schedule generated by a biostatistician using SAS 9.4 software. Patients were randomized into 2 treatment sequence groups: T-R or R-T (where T refers to the test drug, PLD injection manufactured by Shanghai Fudan-Zhangjiang Bio-Pharmaceutical Co., Ltd.; and R refers to the reference drug, Caelyx^®^). The treatments were depicted in **Figure 1**. The dose of PLD for an individual patient was calculated according to body surface area (BSA) using the Mosteller formula for BSA (BSA [m^2^] = ([height (cm) × weight (kg)]/3600)½). The calculated dose of PLD was diluted in 250 mL of 5% dextrose solution for infusion. Then it was administered to patients fasting or 2 hours after a standard non-high-fat breakfast for 90 min ±5 min.

### Safety evaluation

Baseline evaluations would be conducted including complete medical history, physical examination, laboratory measures (hematology, biochemistry, urine analysis, pregnancy test and virology relevant tests), vital signs and physical examination, electrocardiogram (ECG), echocardiogram, brain CT/MRI, and monitoring of adverse events within 30 days of the enrollment and before each treatment. Clinical examination and ECG were repeated on day 1, day 2 and day 14 after the administration of each treatment period. Echocardiogram was repeated 2 weeks and 4 weeks after the administration of each treatment period. Assessment of vital signs, physical examination, adverse events (AEs) and concomitant medication reporting occurred at each visit.

### Blood sample collection and analysis

Blood samples were collected pre-dose, and at 0.25, 0.5, 1.0, 1.5, 1.75, 2, 2.5, 4.5, 6.5, 9.5, 25.5, 49.5, 97.5, 169.5, 241.5, and 337.5 hours after the start of drug infusion in each period. Then the samples were centrifuged within 20 min at 2000 g, 2-8°C for 10 min. The upper plasma was separated, and 1.0ml plasma was transferred to a mixing tube containing 2.0ml stabilizer. After gentle mixing, the blood samples were divided into 5 frozen storage tubes. The plasma samples were transferred to the refrigerator at -80°C for storage within 24 hours.

Plasma concentrations of encapsulated and free doxorubicin were analyzed using two separate validated liquid chromatography mass spectrometry analytical methods (detailed description of bioanalytical methods provided in the reference) (15). Free doxorubicin was pretreated with solid phase extraction (SPE) and encapsulated doxorubicin was pretreated with solid phase extraction (SPE) and protein precipitation, and then analyzed and detected by liquid chromatography tandem mass spectrometry (LC-MS/MS) in positive ion and multi reaction monitoring mode (MRM) with electrospray spray ionization (ESI) as ionization technology.

### Statistical analysis

PK parameters such as AUC_0-t_, AUC_0-∞_, C_max_, elimination half-life (T_1/2_), and time to achieve C_max_ (T_max_) of free and encapsulated doxorubicin were calculated with Phoenix WinNonlin Version 8.3.4 by a non-compartment model, and were analyzed by linear mixed effect model after logarithmic conversion. The experimental results were mainly analysed by descriptive statistics using SAS (SAS Institute, USA, version 9.4). The 90% confidence intervals (CIs) of the geometric mean ratios (GMRs) of C_max_, AUC_0-t_ and AUC_0-∞_ of free and encapsulated doxorubicin and partial AUC of encapsulated doxorubicin between the two formulations were obtained and then converted to the ratio scale by antilog transformation. Bioequivalence would be established if the 90% CIs for the GMRs (test/reference) were completely within the range of 80.00 to 125.00%. The level of significance was set at P <0.05.

Patients with AEs were coded according to the latest version of Medical Dictionary for Regulatory Activities (MedDRA). Severity of AEs was graded according to the National Cancer Institute’s Common Terminology Criteria for Adverse Events (NCI-CTCAE version 5.0).

### Sample size

A two-period crossover design was used in this study. Limited data was available on intra-individual variation (IIV) of PLD pharmacokinetic parameters, so we assumed that the IIV of C_max_ for free doxorubicin was 25%. Considering a 5% probability for type I errors, sample size of 38 patients was required to ensure 80% power to meet bioequivalence. In addition, considering the drop-out/withdrawn rate, 10 patients were added; thus, 48 patients were required for the study.

## Results

### Patient’ demographic characteristics and analysis sets definition

In this study, a total of 66 female patients with advanced breast cancer were screened and assessed for eligibility, and 48 of them were enrolled and randomised. The flowchart of the subject distribution is shown in **Figure 1**. The baseline characteristics of each group are summarized in **Table 1**.

**Table 1:** The Demographic Characteristics.

Parameters	T-R (n=24)	R-T (n=24)	Total (n=48)
Age (y) Mean ± SD	52.0 ± 7.18	50.66 ± 8.62	51.3 ± 7.88
Weight (kg) Mean ± SD	60.98 ± 8.954	62.38 ± 9.196	61.68 ± 9.007
BSA (m^2^) Mean ± SD	1.573 ± 0.13	1.605 ± 0.135	1.589 ± 0.132
ECOG score			
0 n(%)	11(45.8)	14(58.3)	25(52.1)
1 n(%)	13(54.2)	10(41.7)	23(47.9)
Course of disease (month) Mean ± SD	48.465 ± 61.253	27.807 ± 25.499	38.136 ± 47.573
Tumor stage			
IIb n(%)	7(29.2)	7(29.2)	14(29.2)
IIIa n(%)	4(16.7)	2(8.3)	6(12.5)
IIIb n(%)	1(4.2)	4(16.7)	5(10.4)
IIIc n(%)	1(4.2)	0(0)	1(2.1)
IV n(%)	11(45.8)	11(45.8)	22(45.8)

BMI indicates body mass index.

48 patients were randomly and equally assigned to T-R sequence and R-T sequence, with 24 patients in each sequence. In T-R sequence, 2 patients (No. 29 and No.5) experienced AEs (hypersensitivity reaction and infusion reaction) during the administration of the first period, so they withdrew from the trial in the first period and failed to complete the administration according to the requirements of the protocol (the administration time of No. 5 exceeded the time window required by the protocol, and the dosage of No. 29 was 61.4% of the planned dosage). In T-R (No. 30) and R-T (No.12) sequences there was one patient who experienced infusion reaction respectively during the first period of administration. Although these two patients completed the administration of the first period, the administration time exceeded the time window required by the protocol. In addition, both the administration process and concomitant medication in the second period were inconsistent with those in the first period. Because the above conditions may affect the PK results, so the data of the above 4 patients was not included in PK concentration set (PKCS), PK parameter set (PKPS) and bioequivalence set (BES). In T-R sequence, No. 43’s 1.5h data was not included in PKCS in the first period, for the hemolysis degree of the sample was more than 2%, which was beyond the verification range of the methodology, and the free rate (0.871%) was higher than that of other subjects at 1.5h (0.094-0.280%). The concentration of 1.5h sample could not reflect the true concentration. In addition, in R-T sequence, one patient (No. 44) experienced infusion reaction during the first period of administration, and the administration time far exceeded the time range required by the scheme, which may affect the PK results, so the data of the first period was not included in PKCS, PKPS and BES. The definition of analysis sets is shown in the **Table 2**.

**Table 2:** Difinition of Analysis Data Sets.

	T-R	R-T	Total
Screened			66
Enrolled	24	24	48
Completed	22	24	46
FAS	24	24	48
SS	24	24	48
PKCS	21	23	44
PKPS	21	23	44
BES	21	23	44

Full analysis set (FAS). PK concentration set (PKCS). PK parameter set (PKPS). Bioequivalence set (BES). Safety set (SS).

### Pharmacokinetics

Pharmacokinetic analyses were performed on the data from 44 patients. After the administration of the two preparations, the C_max_ and AUC_0-t_ of free doxorubicin were 133.45 ng/ml and 18.72 h*μg/ml, respectively, which were far lower than that of encapsulated doxorubicin, 34.37μg/ml and 3.90 h*mg/ml. Compared with conventional non-liposomal doxorubicin, the t_1/2_ of encapsulated doxorubicin and free doxorubicin released from liposomal were both significantly longer, 81.31 h and 97.31h.The T_max_ of free doxorubicin and liposomal encapsulated doxorubicin was tested by paired rank sum test (Wilcoxon test), and the P values were 0.116 and 0.158, respectively, both greater than 0.05, indicating no significant difference between the two preparations.

**Figure 2:**
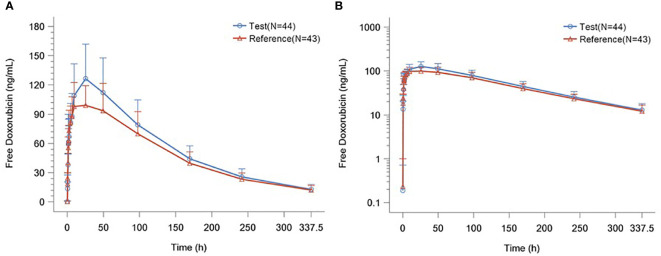
**(A)** Plasma Concentration–time Curves of Free Doxorubicin. **(B)** Plasma Concentration–time Semilogarithmic Curves of Free Doxorubicin.

**Figure 3:**
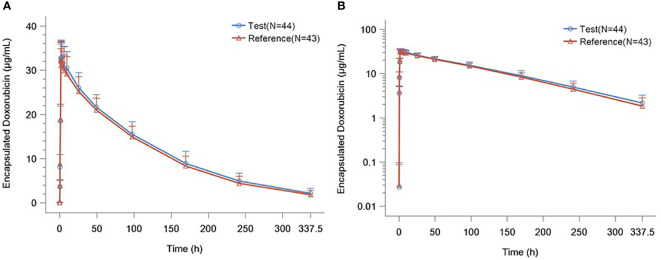
**(A)** Plasma Concentration–time Curves of Liposome Encapsulated Doxorubicin. **(B)** Plasma Concentration–time Semilogarithmic Curves of Liposome Encapsulated Doxorubicin.

The detailed PK parameters of free and encapsulated doxorubicin are presented in **Tables 3** and **4** respectively. The mean plasma concentration-time profiles of free and encapsulated doxorubicin are depicted in **Figures 2A, B** and **Figures 3A, B**.

**Table 3:** The PK Parameters of Free Doxorubicin.

Parameters	Mean ± SD (IIV%)	
	T (N=44)	R (N=43)
C_max_ (ng/mL)	133.45 ± 36.99 (27.7)	114.23 ± 24.14 (21.1)
AUC_0-t_ (h*μg/mL)	18.72 ± 4.89 (26.1)	16.47 ± 3.84 (23.3)
AUC_0-∞_ (h*μg/mL)	20.67 ± 5.36 (25.9)	18.27 ± 4.29 (23.5)
AUC_0-48h_ (h*μg/mL)	5.24 ± 1.28 (24.4)	4.49 ± 0.83 (18.4)
AUC_48h-t_ (h*μg/mL)	13.48 ± 3.80 (28.2)	11.98 ± 3.13 (26.1)
T_max_ (h)	25.5 (1.75, 49.68)	9.5 (1.75, 49.65)
t_1/2_ (h)	97.31 ± 21.21 (21.8)	97.98 ± 19.50 (19.9)
λz (×10^-3^ 1/h)	7.48 ± 1.74 (23.2)	7.36 ± 1.50 (20.4)
Vd (L/m^2^)	358.96 ± 110.66 (30.8)	403.57 ± 113.35 (28.1)
CLz (L/h/m^2^)	2.60 ± 0.77 (29.6)	2.88 ± 0.75 (26.0)

C_max_, maximum concentration; AUC_0-t_ indicates area under the concentration-time curve from 0 to t; AUC_0-∞_, area under the concentration-time curve from 0 to infinity; AUC_0-48h,_ area under concentration-time curve from 0h to 48h; AUC_48h-t_, area under the concentration-time curve from 48h to the last measurable plasma concentration time; T_max_, time to C_max_;

t_1/2_, half-life of elimination; λz, elimination rate constant; V_d_, apparent volume of distribution; CL_Z_ clearance; IIV refers to the intra-individual variation; All data are expressed as arithmetic mean ± standard deviation, except for T_max_, which is expressed as median (min, max).

**Table 4:** The PK Parameters of Encapsulated Doxorubicin.

Parameters	Mean ± SD (IIV%)	
	T (N=44)	R (N=43)
C_max_ (μg/mL)	34.37 ± 3.83 (11.1)	33.73 ± 3.82 (11.3)
AUC_0-t_ (h*mg/mL)	3.90 ± 0.68 (17.3)	3.70 ± 0.60 (16.2)
AUC_0-∞_ (h*mg/mL)	4.19 ± 0.82 (19.7)	3.93 ± 0.74 (18.8)
AUC_0-48h_ (h*mg/mL)	1.26 ± 0.14 (11.2)	1.22 ± 0.13 (11.0)
AUC_48h-t_ (h*mg/mL)	2.64 ± 0.58 (22.1)	2.48 ± 0.51 (20.6)
T_max_ (h)	2.06 (1.5, 9.5)	2.0 (1.5, 9.5)
t_1/2_ (h)	81.31 ± 17.26 (21.2)	78.89 ± 16.57 (21.0)
λz (×10^-3^ 1/h)	8.98 ± 2.30 (25.7)	9.20 ± 2.13 (23.1)
Vd (L/m^2^)	1.41 ± 0.21 (15.1)	1.47 ± 0.30 (20.4)
CLz (mL/h/m^2^)	12.51 ± 3.02 (24.1)	13.16 ± 2.52 (19.2)

C_max_, maximum concentration; AUC_0-t_ indicates area under the concentration-time curve from 0 to t; AUC_0-∞_, area under the concentration-time curve from 0 to infinity; AUC_0-48h,_ area under concentration-time curve from 0h to 48h; AUC_48h-t_, area under the concentration-time curve from 48h to the last measurable plasma concentration time; T_max_, time to C_max_; t_1/2_, half-life of elimination; λz, elimination rate constant; V_d_, apparent volume of distribution; CL_Z_ clearance; IIV refers to the intra-individual variation; All data are expressed as arithmetic mean ± standard deviation, except for T_max_, which is expressed as median (min, max).

### Bioequivalence

A total of 44 patients were included in the BES. The GMRs of the C_max_, AUC_0-t_, AUC_0-∞,_ AUC_0−48h_ and AUC_48h−t_ for free doxorubicin were 114.75%, 112.27%, 111.80%, 115.10%, and 111.06% respectively, and the GMRs of the C_max_, AUC_0-t_, AUC_0-∞,_ AUC_0−48h_ and AUC_48h−t_ for encapsulated doxorubicin were 102.01%, 104.50%, 105.26%, 103.06% and 105.05%, respectively. All 90% confidence intervals (CIs) were within the range of BE required by FDA/EMA and NMPA guidelines (80% to 125%). The results are shown in **Table 5** and **6**.

**Table 5:** The Bioequivalence Analysis of Free Doxorubicin.

Parameters	GM and GMR				IIV %
	T(n=44)	R(n=43)	GMR (%)	90% CI(%)	
C_max_ (ng/mL)	123.74	107.83	114.75	106.25~123.94	21.56
AUC_0-t_ (h*μg/mL)	16.87	15.03	112.27	105.44~119.54	17.47
AUC_0-∞_ (h*μg/mL)	18.52	16.57	111.80	105.17~118.83	16.98
AUC_0-48h_ (h*μg/mL)	4.79	4.16	115.10	108.66~121.92	16.02
AUC_48h-t_ (h*μg/mL)	12.0	10.81	111.06	103.63~119.02	19.29

IIV refers to the intra-individual variation.

GMR refers to the geometric mean ratio of the test over reference pharmacokinetic metric.

**Table 6:** The Bioequivalence Analysis of Liposome Encapsulated Doxorubicin.

Parameters	GM and GMR				IIV %
	T(n=44)	R(n=43)	GMR (%)	90% CI(%)	
C_max_ (ng/mL)	33.60	32.94	102.01	100.26~103.79	4.78
AUC_0-t_ (h*μg/mL)	3.70	3.54	104.50	101.55~107.54	7.91
AUC_0-∞_ (h*μg/mL)	3.93	3.74	105.26	102.11~108.51	8.39
AUC_0-48h_ (h*μg/mL)	1.24	1.20	103.06	100.59~105.58	6.68
AUC_48h-t_ (h*μg/mL)	2.44	2.33	105.05	101.37~108.86	9.85

IIV refers to the intra-individual variation;

GMR refers to the geometric mean ratio of the test over reference pharmacokinetic metric.

### Safety

48 patients were included in the safety set (SS) of the test product (T) and 46 patients were included in the SS of the reference product (R). The incidence of TEAEs related to T and R was 95.8% (46/48) and 97.8% (45/46) respectively, of which the incidence of TEAEs with severity greater than or equal to grade 3 was 6.3% (3/48) and 15.2% (7/46) respectively. There was no significant difference in the incidence and severity of TEAEs between T and R product. The highest incidence of TEAEs was various laboratory abnormalities, and the incidence related to T and R product was 91.7% (44/48) and 84.8% (39/46) respectively. The second was metabolic and nutritional diseases, and the incidence related to T and R product was 58.3% (28/48) and 65.2% (30/46) respectively. In addition, TEAEs with an incidence of more than 20% also include gastrointestinal diseases, anemia, skin and subcutaneous tissue diseases, systemic diseases, various reactions at the administration site, heart organ diseases, etc. There was no significant difference in the incidence of TEAEs between T and R product.

Two patients withdrew from the study due to T-drug-related AEs, including 1 infusion reaction and 1 hypersensitivity reaction with severity of grade 2. There was only 1 serious adverse event (SAE) reported in this study, a grade 3 oral mucositis related to Reference product, which was cured 38 days after the last administration. No deaths occurred during the trial.

Both the two formulation products, T and R, were well-tolerated, and the safety profile was similar. The detailed description of TEAEs was provided in the supplement.

## Discussion

Liposomes have been considered promising and versatile drug vesicles. Compared with traditional drug delivery systems, liposomes exhibit better properties, including site-targeting, sustained or controlled release, protection of drugs from degradation and clearance, superior therapeutic effects, and lower toxic side effects.

The PK of liposome formulation is jointly determined by the PK of carrier and drug release rate. After administered, there are many types of analytes in plasma, including encapsulated drugs and free drugs. Generally speaking, only free drugs are biologically active. For PLD, the same plasma total pharmacokinetics does not mean the same tissue distribution, nor does it mean the same safety and efficacy. So, the BE establishment for PLD does not only rely on a single analyte alone, but on multiple analytes. Initially, the European Medicines Agency (EMA) required to measure the blood concentration of total, free and encapsulated doxorubicin to evaluate the bioequivalence of the generic PLD and the reference drug (16). Since the amount of free doxorubicin is very small, the total amount of doxorubicin is close to the amount of encapsulated doxorubicin. Therefore, current regulatory agencies, including FDA/EMA and China National Medical Products Administration (NMPA), suggest detection of free and encapsulated doxorubicin. In addition, these regulatory principles recommend that the main PK parameters (C_max_ and AUC) of all analytes should meet the BE criteriamay (17–19). Besides, EMA and NMPA recommend the addition of partial AUC (such as AUC_0-48h_ and AUC_48h-t_) measurements of encapsulated doxorubicin as a supportive metric for BE assessment (17, 19). According to the above guidance of regulatory agencies, our results showed that the PLD produced by Shanghai Fudan-Zhangjiang Bio-Pharmaceutical Co., Ltd. has established bioequivalence with Caelyx^®^.

Based on the experience of the regulatory agency, the current BE submission of PLD mostly failed to show BE for the free doxorubicin, but it was unclear that the failure to show BE was caused by large variation in C_max_ or a real difference between products. Studies showed that the IIV of PK parameters of free drugs was much higher than that of encapsulated drugs, especially the IIV of C_max_ was up to 35.5% (14, 20). In addition, a slight change in the release rate of the free doxorubicin from the liposome carrier may cause significant shifts in T_max_ from a few hours to several days, and contribute to the large variation in C_max_ of free doxorubicin. Such variations may probably lead to a non-BE conclusion despite the fact that the drug products were indeed bioequivalent (14). In our study, the IIV of main PK parameters (C_max_ and AUC) of free doxorubicin was also significantly higher than that of encapsulated doxorubicin, and the IIV of C_max_ for free doxorubicin was the highest (21.56%). As IIV of the main PK parameters of free and encapsulated doxorubicin all did not exceed 30%, average bioequivalence (ABE) method was still used to evaluate bioequivalence.

The encapsulated drug can readily be released by external factors including organic solvents, detergents, pH, ionic strength, temperature, mechanical shaking, and freeze-thawing etc which cause the liposome to leak or burst. Due to the instability of liposome formulations in plasma samples, the release of free drug from the liposomal encapsulated doxorubicin during sample handling would result in elevation of measured free doxorubicin concentration (15). Therefore, it is very difficult to accurately determinate the concentration of free doxorubicin in the blood after PLD administration. EMA recommend that in the BE study of PLD the concentrations of free doxorubicin must be achieved by means of appropriate bioanalytical methods rather than by subtracting encapsulated from total drug (17). The bioanalysis for preclinical and clinic studies is a key technical obstacle in the bioequivalence research of liposomes. In our study, we used two separate validated liquid chromatography mass spectrometry analytical methods to analyzed the plasma concentrations of encapsulated and free doxorubicin, meeting the requirements of current regulatory agencies (15).

Although Caelyx^®^ has been in clinical use for over twenty years and been the subject of hundreds of bio-distribution studies, the characterization of its release and uptake profile in tissues and tumor remains incomplete. Encapsulation of drugs in a liposomal nanoparticle can significantly alter its pharmacokinetics and bio-distribution. Direct measurement of liposomal doxorubicin showed that at least 90% of the drug (the assay used cannot quantify less than 5–10% free doxorubicin) remains liposome encapsulated during circulation (13). In contrast to traditional unencapsulated doxorubicin, which displays a large volume of distribution, the small steady state volume of distribution (V_d_) of liposomal encapsulated doxorubicin suggests that PLD is largely confined to vascular fluid, and rarely uptaked by normal tissues (13, 21). Our study shows that the V_d_ of encapsulated doxorubicin of the test product was 1.41 L/m^2^, which was also far lower than that of free doxorubicin, 358.96 L/m^2^. The AUC_0-t_ of free doxorubicin and encapsulated doxorubicin were 18.72 ± 4.89 h*μg/mL and 3.90 ± 0.68 h*mg/mL, respectively. The encapsulated doxorubicin accounted for 99.5% of the total doxorubicin, suggesting that encapsulated doxorubicin was relatively stable in blood circulation with slightly drug released during hemogenization. In addition, according to the results of our study, PLD produced by Shanghai Fudan-Zhangjiang Bio-Pharmaceutical Co., Ltd. showed good PK characteristics of nano liposome formulation, including low rate of clearance (CLz, 12.51 ± 3.02 mL/h/m^2^), high AUC, small V_d_ and a prolonged secondary half-life (t_1/2,_ 81.31 ± 17.26h) that accounts for the majority (> 95%) of the AUC, which was consistent with the reference product.

In our study, we also found that, compared with conventional non-liposomal doxorubicin, the t_1/2_ of free doxorubicin released from liposomes was significantly longer and the CLz was significantly lower, which was consisted with previous literature (14). This may be due to the slow release of free doxorubicin from the liposome, whose release rate is apparently lower than the clearance rate.

In addition to PK-BE, the two formulation products were well tolerated, and the safety profile was similar.

## Conclusion

In this study, the C_max_, AUC_0-t_, and AUC_0-∞_ values for both free doxorubicin and liposome encapsulated doxorubicin were comparable between the test and the reference products. The 90% CIs for C_max_ and AUC ratios all fell within the acceptable range of 80 to 125%, supporting the claim that PLD injection developed by Shanghai Fudan-Zhangjiang Bio-Pharmaceutical Co., Ltd. was bioequivalent to Caelyx^®^ in patients with advanced breast cancer. Both the test and the reference formulations were well-tolerated, and the safety profile was similar.

## Data availability statement

The original contributions presented in the study are included in the article/**Supplementary Material**. Further inquiries can be directed to the corresponding authors.

## Ethics statement

The studies involving human participants were reviewed and approved by the institutional ethics committee of Beijing Shijitan Hospital affiliated to Capital Medical University. The patients/participants provided their written informed consent to participate in this study.

## Author contributions

XW, YPL and YJL had involved in study design, protocol review, study conduct, drafting the manuscript and revising critically for important intellectual content. All authors contributed to the article and approved the submitted version.
