# Supplementary material for: A multicenter randomized trials to compare the bioequivalence and safety of a generic doxorubicin hydrochloride liposome injection with Caelyx ® in advanced breast cancer
Source: Front Oncol. 2022 Dec 20;12:1070001. doi: 10.3389/fonc.2022.1070001 (PMC9810385; doi:10.3389/fonc.2022.1070001)
Supplement: Supplementary file 1 [file Table_1.docx]

Table 1. Summary of Treatment-emergent Adverse Events (TEAE) (SS)

|  | T | R | Total |
| --- | --- | --- | --- |
| **N** | 48 | 46 | 48 |
| **Total n(%)** | 46(95.8) | 45(97.8) | 48(100.0) |
| **All kinds of investigations n(%)** | 44(91.7) | 39(84.8) | 44(91.7) |
| Leukopenia n(%) | 22(45.8) | 25(54.3) | 27(56.3) |
| Leukopenia n(%) | 16(33.3) | 18(39.1) | 25(52.1) |
| Monocyte count decreased n(%) | 7(14.6) | 10(21.7) | 13(27.1) |
| Low lymphocyte count n(%) | 10(20.8) | 12(26.1) | 13(27.1) |
| Urine leucocyte positive n(%) | 9(18.8) | 4(8.7) | 12(25.0) |
| QT interval prolonged n(%) | 9(18.8) | 9(19.6) | 11(22.9) |
| Thrombocytopenia n(%) | 7(14.6) | 9(19.6) | 10(20.8) |
| Blood alkaline phosphatase increased n(%) | 4(8.3) | 4(8.7) | 6(12.5) |
| Elevated blood glucose n(%) | 2(4.2) | 4(8.7) | 6(12.5) |
| Elevated blood lactate dehydrogenas n(%) | 3(6.3) | 4(8.7) | 6(12.5) |
| γ-glutamyltransferase increased n(%) | 2(4.2) | 5(10.9) | 5(10.4) |
| Increased cerebral diuretic natriuretic peptide n(%) | 4(8.3) | 2(4.3) | 5(10.4) |
| Urine leucocyte esterase positive n(%) | 4(8.3) | 3(6.5) | 5(10.4) |
| Elevated D-dimer n(%) | 3(6.3) | 2(4.3) | 5(10.4) |
| Abnormal T wave  n(%) | 3(6.3) | 2(4.3) | 5(10.4) |
| Elevated serum creatinine n(%) | 1(2.1) | 4(8.7) | 5(10.4) |
| Elevated fibrinogen n(%) | 2(4.2) | 3(6.5) | 4(8.3) |
| Elevated alanine aminotransferase n(%) | 1(2.1) | 2(4.3) | 3(6.3) |
| Elevated aspartate aminotransferase n(%) | 2(4.2) | 1(2.2) | 3(6.3) |
| Elevation of blood bilirubin n(%) | 1(2.1) | 1(2.2) | 2(4.2) |
| Blood iron reduced n(%) | 0(0) | 2(4.3) | 2(4.2) |
| Elevated platelet count n(%) | 1(2.1) | 2(4.3) | 2(4.2) |
| Elevated alpha-hydroxybutyrate dehydrogenase n(%) | 1(2.1) | 0(0) | 1(2.1) |
| Elevated white blood cell count n(%) | 1(2.1) | 0(0) | 1(2.1) |
| Elevated conjugated bilirubin n(%) | 1(2.1) | 0(0) | 1(2.1) |
| Urine occult blood positive n(%) | 0(0) | 1(2.2) | 1(2.1) |
| Physiological weight-decreased n(%) | 1(2.1) | 0(0) | 1(2.1) |
| PR interval prolonged n(%) | 1(2.1) | 0(0) | 1(2.1) |
| Electrocardiogram high voltage n(%) | 0(0) | 1(2.2) | 1(2.1) |
| Heart rate decreased n(%) | 1(2.1) | 1(2.2) | 1(2.1) |
| Heart rate increased n(%) | 0(0) | 1(2.2) | 1(2.1) |
| Decreased blood albumin n(%) | 0(0) | 1(2.2) | 1(2.1) |
| Elevated serum creatine phosphokinase n(%) | 0(0) | 1(2.2) | 1(2.1) |
| Elevated blood pressure n(%) | 1(2.1) | 0(0) | 1(2.1) |
| Elevated neutrophil count n(%) | 1(2.1) | 0(0) | 1(2.1) |
| **Metabolic and nutritional diseases n(%)** | 28(58.3) | 30(65.2) | 37(77.1) |
| Hypercholesterolemia n(%) | 9(18.8) | 9(19.6) | 16(33.3) |
| Hypertriglyceridemia n(%) | 14(29.2) | 8(17.4) | 16(33.3) |
| Anorexia n(%) | 6(12.5) | 8(17.4) | 12(25.0) |
| Hyperuricemia n(%) | 3(6.3) | 7(15.2) | 10(20.8) |
| Hyperglycosemia n(%) | 5(10.4) | 4(8.7) | 7(14.6) |
| Hypoproteinemia n(%) | 2(4.2) | 5(10.9) | 6(12.5) |
| Hyponatremia n(%) | 3(6.3) | 5(10.9) | 6(12.5) |
| Hypokalemia n(%) | 0(0) | 1(2.2) | 1(2.1) |
| Hypochloremia n(%) | 0(0) | 1(2.2) | 1(2.1) |
| VitaminB1Deficiency n(%) | 1(2.1) | 0(0) | 1(2.1) |
| **Gastrointestinal system disorders n(%)** | 24(50.0) | 27(58.7) | 35(72.9) |
| Nause n(%) | 14(29.2) | 15(32.6) | 20(41.7) |
| Oral mucositis n(%) | 10(20.8) | 12(26.1) | 15(31.3) |
| Vomit n(%) | 8(16.7) | 7(15.2) | 12(25.0) |
| Oral ulcer n(%) | 4(8.3) | 5(10.9) | 8(16.7) |
| Oesophagitis n(%) | 4(8.3) | 5(10.9) | 7(14.6) |
| Belching n(%) | 3(6.3) | 3(6.5) | 4(8.3) |
| Flatulence n(%) | 3(6.3) | 0(0) | 3(6.3) |
| Thirst n(%) | 0(0) | 3(6.5) | 3(6.3) |
| Constipation n(%) | 2(4.2) | 2(4.3) | 2(4.2) |
| Abdominal distension n(%) | 2(4.2) | 0(0) | 2(4.2) |
| Abdominal pain n(%) | 1(2.1) | 1(2.2) | 2(4.2) |
| Diarrhea n(%) | 1(2.1) | 0(0) | 1(2.1) |
| **Blood and lymphatic system disorders n(%)** | 10(20.8) | 9(19.6) | 16(33.3) |
| Anemia n(%) | 10(20.8) | 9(19.6) | 16(33.3) |
| **Skin and subcutaneous tissue disorders n(%)** | 8(16.7) | 7(15.2) | 14(29.2) |
| Hyperpigmentation n(%) | 5(10.4) | 5(10.9) | 10(20.8) |
| Rash n(%) | 1(2.1) | 3(6.5) | 4(8.3) |
| Pruritus n(%) | 2(4.2) | 1(2.2) | 3(6.3) |
| - Eczema n(%) | 2(4.2) | 0(0) | 2(4.2) |
| - Skin peeling n(%) | 0(0) | 1(2.2) | 1(2.1) |
| - Skin ulcer n(%) | 1(2.1) | 0(0) | 1(2.1) |
| - Dermatitis n(%) | 0(0) | 1(2.2) | 1(2.1) |
| - Drug eruption n(%) | 0(0) | 1(2.2) | 1(2.1) |
| Metacarpus red swelling syndrome n(%) | 1(2.1) | 0(0) | 1(2.1) |
| - Nail Discolouration n(%) | 0(0) | 1(2.2) | 1(2.1) |
| **General disorders and administration site conditions n(%)** | 5(10.4) | 10(21.7) | 12(25.0) |
| - Weak n(%) | 5(10.4) | 7(15.2) | 9(18.8) |
| - Fever n(%) | 0(0) | 1(2.2) | 1(2.1) |
| - Facial swelling n(%) | 0(0) | 1(2.2) | 1(2.1) |
| - Peripheral swelling n(%) | 0(0) | 1(2.2) | 1(2.1) |
| **Cardiac disorders n(%)** | 7(14.6) | 8(17.4) | 12(25.0) |
| - Sinus tachycardia n(%) | 2(4.2) | 4(8.7) | 5(10.4) |
| - Sinus bradycardia n(%) | 1(2.1) | 2(4.3) | 2(4.2) |
| Sinus arrhythmia n(%) | 1(2.1) | 1(2.2) | 2(4.2) |
| Supraventricular anterior contraction n(%) | 1(2.1) | 0(0) | 1(2.1) |
| Tachycardia n(%) | 1(2.1) | 1(2.2) | 1(2.1) |
| - Palpitation n(%) | 0(0) | 1(2.2) | 1(2.1) |
| - Arrhythmia n(%) | 1(2.1) | 0(0) | 1(2.1) |
| - The heart discomfort n(%) | 1(2.1) | 0(0) | 1(2.1) |
| - Left atrial dilatation n(%) | 1(2.1) | 0(0) | 1(2.1) |
| **Various injuries, poisoning and operation complications n(%)** | 5(10.4) | 7(15.2) | 11(22.9) |
| Infusion related reaction n(%) | 5(10.4) | 7(15.2) | 11(22.9) |
| **Musculoskeletal and connective tissue disorders n(%)** | 5(10.4) | 5(10.9) | 8(16.7) |
| - Joint pain n(%) | 1(2.1) | 1(2.2) | 2(4.2) |
| - Limb pain n(%) | 1(2.1) | 2(4.3) | 2(4.2) |
| - Back pain n(%) | 1(2.1) | 0(0) | 1(2.1) |
| - Myalgia n(%) | 1(2.1) | 1(2.2) | 1(2.1) |
| - Neck pain n(%) | 0(0) | 1(2.2) | 1(2.1) |
| - Musculoskeletal pain in the chest n(%) | 1(2.1) | 0(0) | 1(2.1) |
| **Nervous system disorders n(%)** | 5(10.4) | 4(8.7) | 6(12.5) |
| - Headache n(%) | 2(4.2) | 3(6.5) | 3(6.3) |
| - Dizzy n(%) | 3(6.3) | 1(2.2) | 3(6.3) |
| - Drowsiness n(%) | 1(2.1) | 0(0) | 1(2.1) |
| - Parageusi n(%) | 0(0) | 1(2.2) | 1(2.1) |
| **Diseases of the hepatobiliary system n(%)** | 4(8.3) | 2(4.3) | 4(8.3) |
| - Abnormal liver function n(%) | 3(6.3) | 1(2.2) | 3(6.3) |
| - Hyperbilirubinemia n(%) | 1(2.1) | 1(2.2) | 2(4.2) |
| **Infectious diseases n(%)** | 3(6.3) | 1(2.2) | 4(8.3) |
| - Urinary tract infection n(%) | 1(2.1) | 1(2.2) | 2(4.2) |
| Bronchitis n(%) | 1(2.1) | 0(0) | 1(2.1) |
| - Tinea pedis n(%) | 1(2.1) | 0(0) | 1(2.1) |
| **Benign, malignant, and unknown nature of tumors n(%)** | 2(4.2) | 1(2.2) | 3(6.3) |
| Cancer pain n(%) | 2(4.2) | 1(2.2) | 3(6.3) |
| **Kidney and urinary system disorders n(%)** | 1(2.1) | 2(4.3) | 3(6.3) |
| Haematuria n(%) | 0(0) | 2(4.3) | 2(4.2) |
| - Proteinuria n(%) | 1(2.1) | 0(0) | 1(2.1) |
| **Respiratory, thoracic and mediastinal disorders n(%)** | 1(2.1) | 2(4.3) | 2(4.2) |
| - Cough n(%) | 0(0) | 1(2.2) | 1(2.1) |
| Oropharyngeal pain n(%) | 1(2.1) | 1(2.2) | 1(2.1) |
| **Vascular disorders n(%)** | 2(4.2) | 1(2.2) | 2(4.2) |
| - Hypertension n(%) | 2(4.2) | 1(2.2) | 2(4.2) |
| **Ear and labyrinth disorders n(%)** | 1(2.1) | 0(0) | 1(2.1) |
| Earache n(%) | 1(2.1) | 0(0) | 1(2.1) |
| **Psychotic disease n(%)** | 0(0) | 1(2.2) | 1(2.1) |
| Insomnia n(%) | 0(0) | 1(2.2) | 1(2.1) |
| - **Immune system disorders n(%)** | 1(2.1) | 0(0) | 1(2.1) |
| - Hypersensitivity reaction n(%) | 1(2.1) | 0(0) | 1(2.1) |
